# The diversity of unique 1,4,5,6-Tetrahydro-2-methyl-4-pyrimidinecarboxylic acid coding common genes and Universal stress protein in Ectoine TRAP cluster (UspA) in 32 *Halomonas* species

**DOI:** 10.1186/s13104-021-05689-3

**Published:** 2021-08-03

**Authors:** Bhagwan Narayan Rekadwad, Wen-Jun Li, P. D. Rekha

**Affiliations:** 1 Yenepoya Research Centre, Yenepoya (Deemed to be University), University Road, Deralakatte, Mangalore, Karnataka 575018 India; 2 State Key Laboratory of Biocontrol, Guangdong Provincial Key Laboratory of Plant Resources and Southern Marine Science and Engineering Guangdong Laboratory (Zhuhai), School of Life Sciences, Sun Yat-Sen University, Guangzhou, 510275 China

**Keywords:** Ectoine, Life under extreme conditions, Saline environments, Single-copy genes, Ancient bacteria and Archaea, Bioactive compounds

## Abstract

**Objectives:**

To decipher the diversity of unique ectoine-coding housekeeping genes in the genus *Halomonas*.

**Results:**

In *Halomonas*, 1,4,5,6-Tetrahydro-2-methyl-4-pyrimidinecarboxylic acid has a crucial role as a stress-tolerant chaperone, a compatible solute, a cell membrane stabilizer, and a reduction in cell damage under stressful conditions. Apart from the current 16S rRNA biomarker, it serves as a blueprint for identifying *Halomonas* species. *Halomonas elongata* 1H9 was found to have 11 ectoine-coding genes. The presence of a superfamily of conserved ectoine-coding among members of the genus *Halomonas* was discovered after genome annotations of 93 *Halomonas* spp. As a result of the inclusion of 11 single copy ectoine coding genes in 32 *Halomonas* spp., genome-wide evaluations of ectoine coding genes indicate that 32 *Halomonas* spp. have a very strong association with *H. elongata* 1H9, which has been proven evidence-based approach to elucidate phylogenetic relatedness of ectoine-coding child taxa in the genus *Halomonas*. Total 32 *Halomonas* species have a single copy number of 11 distinct ectoine-coding genes that help *Halomonas* spp., produce ectoine under stressful conditions. Furthermore, the existence of the Universal stress protein (UspA) gene suggests that *Halomonas* species developed directly from primitive bacteria, highlighting its role during the progression of microbial evolution.

**Supplementary Information:**

The online version contains supplementary material available at 10.1186/s13104-021-05689-3.

## Introduction

1,4,5,6-Tetrahydro-2-methyl-4-pyrimidinecarboxylic acid (C_6_H_10_N_2_O_2_, molecular weight 142.16) is a natural pigment produced within the cytoplasm of salt-loving bacteria (e.g. genus *Ectothiorhodospira*; *Halomonas*). This pigment helps bacterium to perform osmoregulatory function termed as ‘ectoine’ in general [6]. The moderately halophilic members of family *Halomonadaceae* displays osmoadaptation facilitated by pigments such as betaine and ectoine [3] and hydroxyectoine [12]. Family *Halomonadaceae* possess total 18 child taxa. Of these, names of 14 child taxa are validly published with their correct name. Other16 child taxa have their validly published name including synonyms under the International Code of Nomenclature of Prokaryotes (ICNP). On similar note, currently Genus *Halomonas* represented by 114 type strains with 112 candidates having validity published name and correct name and 10 candidates with synonyms. Also, three species have orthographic misspelled variants, and 18 invalidated species were not validated by ICNP [7]. Description of all *Halomonas* species is given on LPSN portal managed by Leibniz Institute DSMZ-German Collection of Microorganisms and Cell Cultures GmbH, Germany. *Halomonas* species are known producer of biotechnologically important ectoine. Being suspended in the cytoplasm, ectoine and hydroxyectoine coded by *Halomonas* species has benefits to cell. It performs various activities in cell such as stress tolerant chaperones, as a compatible solute, stabilize of cell membrane and reduce cell damage [10]. Moreover, ectoine and hydro-ectoines are high-value chemicals and exploited for cosmetics, immune protection, stabilization of antibodies, anti-inflammatory and tissue protective agent, for co-production of bioplastic polyhydroxybutyrate [8], as a skin aging and protectant agent against harsh environments viz. radiation and extreme temperatures. Whole cell and macromolecule under hostile conditions were protected by intracellular ectoine from freezing, drying, high salinity, heat stress, oxygen radicals, radiation and denaturing agents [10]. Various applications of ectione produced by *Halomonas* species reflect presence of diverse gene profiles and other conserved genes in their genomes. It is therefore important to evaluate indicative signatures genes that codes ectoine and governs vital biological function under extreme environmental conditions among the genus *Halomonas*.

Present study is a blue print of ectoine coding genes identified from *H. elongata.* Genome annotations of existing *Halomonas* spp., have uncovered existence of some common genes that codes ectoine (s) among members of the genus *Halomonas*. Thus, genome-wide evaluations of ectoine coding genes were assessed. We also analyzed highly close 32 *Halomonas* spp., with *Halomonas elongata* 1H9, which has phylogenetic related ectoine coding child taxa inferred using identified single copy genes.

## Main text

### Methods

#### 128 type strains 16S rRNA genes and 94 *Halomonas* spp., genomes

One hundred twenty-eight 16S rRNA genes of type strains and 94 complete genomes and reference sequences of *Halomonas* spp., were obtained from LPSN and NCBI genome database deposited during 2006 to 2020.

#### Radar chart

*Halomonas* spp., possesses multiple quantitative variables (species in particular) i.e. variable genome length/data points for visualization. Radar chart makes the way easy to compare the intra-species variable length to see similar values and find high or low scoring within outliers in the genus.

#### RAST genome analysis

Complete genome sequences of *Ectothiorhodospira haloalkaliphila* ATCC 51935 (CP007268), *H. elongata* 1H9 (NC_014532), *Halorhodospira halochloris* DSM 1059 (AP017372) and *Halorhodospira halophila* SL1 (CP000544) analyses done using RAST v2.0 (https://rast.nmpdr.org/) [11]. RAST server is a SEED-based National Microbial Pathogen Database Resource (NMPDR), prokaryotic genome annotation service, to predict system coverage, subsystem category distribution and subsystem feature count [2].

#### Identification of protein families and single copy genes

Protein families and single-copy genes in 93 *Halomonas* spp., were identified using PATRIC 3.6.9 (https://www.patricbrc.org/). PLfams within the genus were computed with MCL inflation = 3.0 to obtain higher sequence similarity and better specificity for intra-genus/species close comparisons.

#### Selecting single copy number genes

PLfams of 1,4,5,6-Tetrahydro-2-methyl-4-pyrimidinecarboxylic acid coding genes among 93 *Halomonas* spp., were extracted. Common genes coded by *Halomonas* species were selected for analysis. The topology of the phylogenetic tree generated using concatenated sequences was compared with the topology of 16S rRNA based *Halomonas* spp., child taxa tree.

#### Phylogeny reconstruction and topology analysis

The evolutionary history of one hundred twenty-eight16S rRNA and 33 *Halomonas* single-copy genes were inferred using standalone tool MEGA X with 1000 bootstrap analysis followed by best scoring ML, NJ and ME tree. The Jukes-Cantor method and are in the units of the number of base substitutions per site. The closest child taxa of biotechnological important ectoine producing *H. elongata* 1H9 were deciphered. It helps for phylogenetic analysis and topology comparison to delineate nearest species and 1,4,5,6-Tetrahydro-2-methyl-4-pyrimidinecarboxylic acid gene coding species.

### Results

#### Phylogenetic analysis of 16S rRNA genes in the genus *Halomonas*

*H. elongata* 1H9 is a bacterium that prefers saline environment and known for 1,4,5,6-Tetrahydro-2-methyl-4-pyrimidinecarboxylic acid (ectoine) producer under extreme environmental condition.

RAST genome analysis of the *H. elongata* 1H9 shows that various subsystem feature consists of various pathways (Additional file 1: Figure S1) coded by bacterium. In addition, member of the genus *Halomonas* encodes and produce molecular variants of 1,4,5,6-Tetrahydro-2-methyl-4-pyrimidinecarboxylic acid. Therefore, the diversity of ectoine coding *Halomonas* might form distinct cluster with a similar kind of *Halomonas* species. Hence, phylogenetic analysis of 16S rRNA sequences of type strain amongst genus *Halomonas* revealed that type strains AJ261, 1H9, M8, 5-3, RS-16, AAD6, SS20, 11S, NTU-107, TBZ21, 5CR, F8-11, SL014B-69, TBZ202, KCTC 42685, Z-7009, SL014B-85, CIP 105456, 204, KMM 1376, 10-C-3, Hwa etc., (Additional file 2: Figure S2) formed a discrete clustered together from extracted sequences. This suggests that those species have a similar gene pool regardless of their genome length were grouped in one cluster. Variation in some branches may occur due to the use of single 16S rRNA genes for phylogenetic analysis. Hence, members of the genus *Halomonas* might possess similar single-copy ectoine coding genes reveals that apart from the 16S RNA gene.

#### Identification of protein families, single copy genes and Pearson correlation

**Fig. 1:**
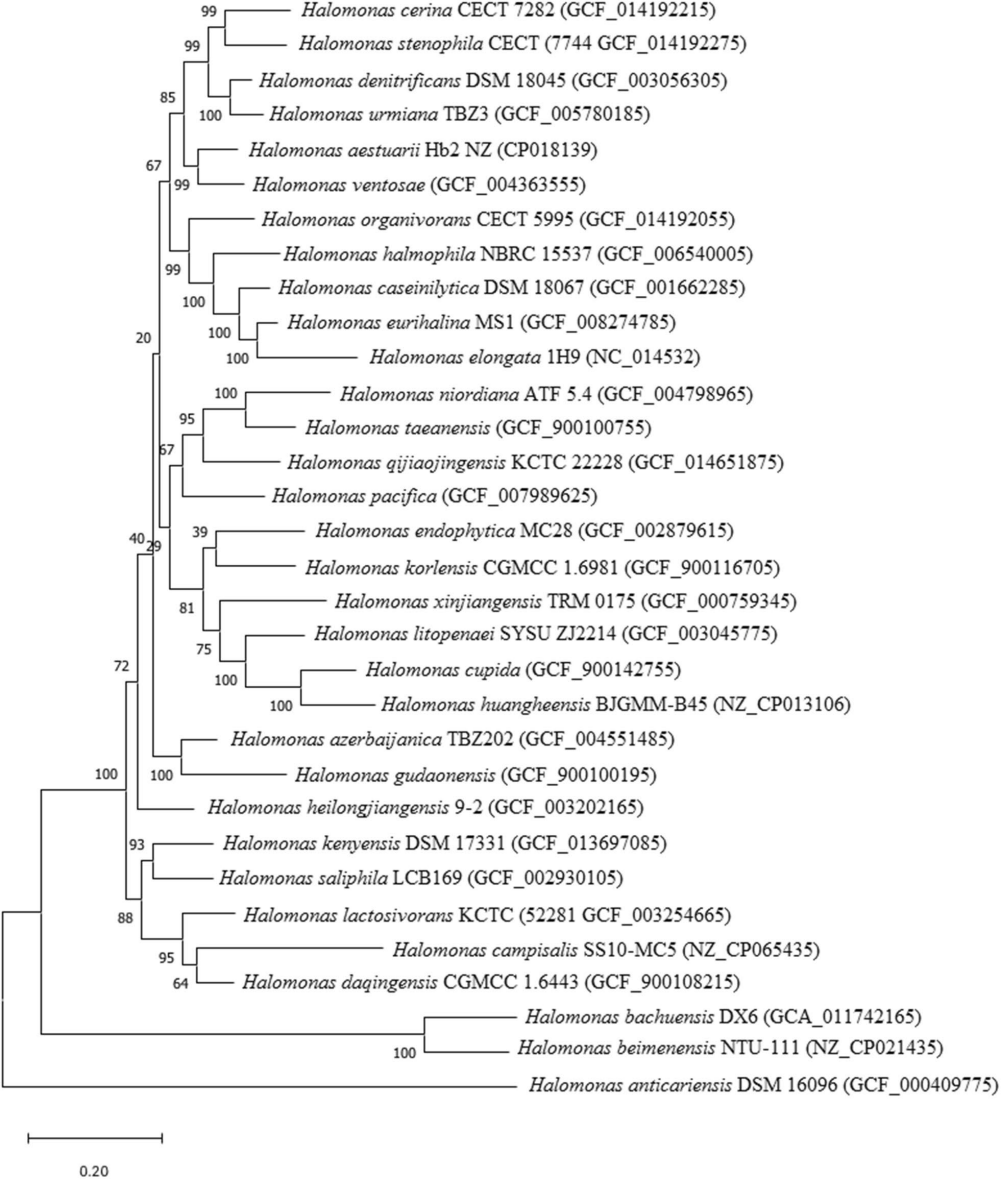
Maximum-likelihood (ML) analysis of concatenated sequences of 11 genes (DoeA-DoeC-DoeX-EctC-EctD-EutB-EutC-TeaA-TeaB-TeaC-UspA) from 32 *Halomonas* species in MEGA X. The evolutionary distances were computed using the Jukes-Cantor method and are in the units of the number of base substitutions per site. The percentage of replicate trees in which the associated taxa clustered together in the bootstrap test (1000 replicates)

**Fig. 2:**
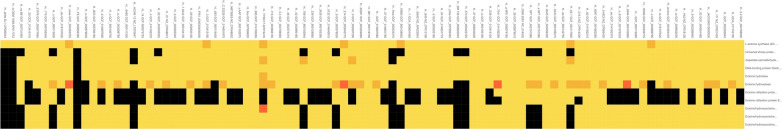
Heatmap of 11 ectoine coding genes in *Halomonas* spp., showing genome and protein-pairwise average linkage using Pearson correlation. Heat map has been inferred from annotated genes among the genera *Halomonas* under investigation in this study

Whole-genome analyses and annotation have resolved the misery of unique genes distributed among the genus *Halomonas* spp. The radar chart shows that existing genomic data of *Halomonas* spp., possesses complete genome sequences, reference genomes and some scaffolds (Additional file 3: Figure S3, Additional File 6: Table S1). Available genomic sequence data shows a similar gene pool and all ectoine-coding sequences from 93 type strains not having sets of genes. To resolve this issue and find relevant species in the genus *Halomonas*, we, therefore, annotated all genomes and identified the single-copy gene that codes ectoine. It was noticed that few *Halomonas* species that more than 11 single copy ectoine-coding genes. Therefore, inferred ML tree (Additional file 4: Figure S4) some type strains shows that ectoine biomarker (in 1H9, F9-6, AJ261, SP4, ACAM 71, 62, Hb3, DSM 15,911, N12, NTU-107, G-16.1, ZJ2214, TBZ3, M29, 79, BJGMM-B45, LCB169, CFH 9008, AIR-2, DQD2-30, 4A, SL014B-69, TBZ202, DX6, 9-2 and MC28) possessed by species were more or less similar kind of representative species similar to concatenated sequence of 32 *Halomonas* species (Fig. 1). It was observed that of the 93 annotated genome sequences, 31 + 1 (32) species have 11 ectoine coding genes (DoeA-DoeC-DoeX-EctC-EctD-EutB-EutC-TeaA-TeaB-TeaC-UspA) as single copy number genes (Additional file 5: Figure S5; Table 1). Heatmap of 11 ectoine coding genes shows a high degree of Pearson correlation (Fig. 2) value lies between 0.50 and ± 1 (0 = no correlation, 1 = high degree correlation).

**Table 1:** Functions of ectoine-coding genes in the Genus *Halomonas* under different scenarios

Gene symbol	Gene description/accepted name	EC number	KEGG Id (KO) & name	Gene name in patric server	Comment/involved in pathways & KEGG KO Id
DoeA	Ectoine hydrolase DoeA	3.5.4.44	–	Ectoine hydrolase	The enzyme, found in some halophilic bacteria, is involved in the degradation of the compatible solute ectoine The enzyme, which belongs to peptidase family M24, only acts in the direction of ectoine hydrolysis It also produces smaller amounts of (2S)-4-acetamido-2-aminobutanoate, which is recycled back to ectoine by EC 4.2.1.108
DoeC	Aspartate-semialdehyde dehydrogenase	1.2.1.11	–	Aspartate-semialdehyde dehydrogenase DoeC in ectoine degradation	Threonine and Homoserine Biosynthesis Lysine Biosynthesis DAP Pathway, GJO scratch Lysine Biosynthesis DAP Pathway
DoeX	DNA-binding protein DoeX, ectoine utilization regulator	No EC recorded	K15782 & Lrp/AsnC family transcriptional regulator, regulator of ectoine-degradation genes	DNA-binding protein DoeX, ectoine utilization regulator	Glycine, serine and threonine metabolism Monobactam biosynthesis Cysteine and methionine metabolism Lysine biosynthesis Metabolic pathways Biosynthesis of secondary metabolites Microbial metabolism in diverse environments
EctC	Ectoine synthase	4.2.1.108	–	l-Ectoine synthase (EC 4.2.1.108)	Ectoine is an osmoprotectant that is found in halophilic eubacteria This enzyme is part of the ectoine biosynthesis pathway and only acts in the direction of ectoine formation
EctD	Ectoine hydroxylase	1.14.11.55	–	Ectoine hydroxylase	The enzyme, found in bacteria, is specific for ectoine Glycine, serine and threonine metabolism Metabolic pathways
EutB	Ethanolamine ammonia-lyase large subunit	4.3.1.7	–	Ectoine utilization protein EutB, threonine dehydratase-like	Glycerophospholipid metabolism Metabolic pathways
EutC	Ethanolamine ammonia-lyase small subunit	4.3.1.7	–	Ectoine utilization protein EutC, similar to ornithine cyclodeaminase	Glycerophospholipid metabolism Metabolic pathways
TeaA	TRAP transporter substrate-binding protein	No EC recorded	K11688 & C4-dicarboxylate-binding protein (DctP)	Ectoine/hydroxyectoine TRAP transporter substrate-binding periplasmic protein TeaA	–
TeaB	Ectoine/hydroxyectoine TRAP transporter small permease protein	No EC recorded	–	Ectoine/hydroxyectoine TRAP transporter small permease protein TeaB	–
TeaC	Ectoine/hydroxyectoine TRAP transporter large permease protein	No EC recorded	K11690 C4-dicarboxylate transporter, DctM subunit	Ectoine/hydroxyectoine TRAP transporter large permease protein TeaC	–
UspA	Universal stress protein A	No EC recorded		Universal stress protein UspA in Ectoine TRAP cluster	–

Table represents accepted designations and codes ectoine coding genes along with their alternative names and applications/performed reactions in microorganisms

#### Novel Universal stress protein in Ectoine TRAP cluster (UspA) and resistance mediated by UspA gene

Studies on genome sequence analyses and analysis of various ectoine coding in *Halomonas* spp., uncovered that type strains viz. *H. aestuarii* Hb2 (NZ_CP018139), *H. anticariensis* DSM 16096 (GCF_000409775), *H. azerbaijanica* TBZ202 (GCF_004551485), *H. bachuensis* DX6 (GCA_011742165), *H. beimenensis* NTU-111 (NZ_CP021435), H. campisalis SS10-MC5 (NZ_CP065435), *H. caseinilytica* DSM 18067 (GCF_001662285), *H. cerina* CECT 7282 (GCF_014192215), *H. cupida* (GCF_900142755), *H. daqingensis* CGMCC 1.6443 (GCF_900108215), *H. denitrificans* DSM 18,045 (GCF_003056305), *H. endophytica* MC28 (GCF_002879615), *H. eurihalina* MS1 (GCF_008274785), *H. gudaonensis* (GCF_900100195), *H. halmophila* NBRC 15537 (GCF_006540005), *H. heilongjiangensis* 9-2 (GCF_003202165), *H. huangheensis* BJGMM-B45 (NZ_CP013106), *H. kenyensis* DSM 17331 (GCF_013697085), *H. korlensis* CGMCC 1.6981 (GCF_900116705), *H. lactosivorans* KCTC 52281 (GCF_003254665), *H. litopenaei* SYSU ZJ2214 (GCF_003045775), *H. niordiana* ATF 5.4 (GCF_004798965), *H. organivorans* CECT 5995 (GCF_014192055), *H. pacifica* (GCF_007989625), *H. qijiaojingensis* KCTC 22228 (GCF_014651875), *H. saliphila* LCB169 (GCF_002930105), *H. stenophila* CECT 7744 (GCF_014192275), *H. taeanensis* (GCF_900100755), *H. urmiana* TBZ3 (GCF_005780185), *H. ventosae* (GCF_004363555), *H. xinjiangensis* TRM 0175 (GCF_000759345) and *H. zincidurans* B6 (GCF_000731955) possess superfamily of conserved gene—UspA—suggests that the UspA gene/domain has been inherited from ancient protein family found in primitive bacteria. UspA protein helps *Halomonas* species provide support and assist *Halomonas* to function and produce ectoine in the saline environment under stressful conditions like high salt, low water activity and low temperature etc. Hence, UspA—stress protein—found in 32 species is a new report in the genus *Halomonas*.

Moreover, ectoine or ectoine derivatives investigated by various groups worldwide for their biotechnological applications. For instance, few reports suggests that ectoine or ectoine derivatives were been in use for oral care, vulvovaginal conditions and in some in cosmetic formulations to protect cell damage and avoid microbial infections. For instance, reports suggest that ectoine and ectoin derivatives in combination with natural essential oil were employed as effective solution against pathogenic *Pseudomonas aeruginosa* [1] and antifungal resistant *Candida* strains causing candidiasis [4, 5]. Therefore, in biotechnological perspectives ectoine and derivatives of ectoines may have application against antimicrobial resistance and multi-drug resistant microorganisms.

### Conclusion

Ectoine signatures can be found in 93 *Halomonas* genome sequences that are publicly available. 32 *Halomonas* species have 11 separate ectoine genes in a single copy number in their genomes, which help *Halomonas* spp. produce ectoine under stressful conditions. Based on existing genomic data, it was discovered that *H. elongata* 1H9 has distinct ectoine-producing machinery from other *Halomonas* species. The existence of 11 distinct genes in 32 species, including the UspA gene, suggests that *Halomonas* species evolved directly from their primitive ancestor, shedding light on their evolutionary significance.

## Limitations

A possible restriction would be the presence of biomarkers other than existing ectoine-coding genes responsible for *Halomonas* spp. producing 1,4,5,6-Tetrahydro-2-methyl-4-pyrimidinecarboxylic acid.

## Supplementary Information

**Additional file 1: Figure S1.** RAST genome analysis of *H. elongata* 1H9 indicates subsystem coverage and distributed subsystem features in annotated genome. Each subsystem feature possesses pathways encoded by respective genes.

**Additional file 2: Figure S2.** The evolutionary history of *Halomonas* species was inferred using the Neighbor-Joining method. Analysis using 16S rRNA gene sequences were conducted in MEGA X. The evolutionary distances were computed using the Jukes-Cantor method and are in the units of the number of base substitutions per site. The percentage of replicate trees in which the associated taxa clustered together in the bootstrap test (1000 replicates).

**Additional file 3: Figure S3.** RADAR Chart of Genus *Halomonas* spp. (see supplementary table F1 for names of the species). Yellow circle indicates average genome length of each species and differences in genome length.

**Additional file 4: Figure S4.** Maximum-likelihood analysis among *Halomonas* species was inferred from 16S rRNA gene sequences in MEGA X. The evolutionary distances were computed using the Jukes-Cantor method and are in the units of the number of base substitutions per site. The percentage of replicate trees in which the associated taxa clustered together in the bootstrap test (1000 replicates).

**Additional file 5: Figure S5.** Single copy ectoine coding genes (DoeA-DoeC-DoeX-EctC-EctD-EutB-EutC-TeaA-TeaB-TeaC-UspA) in the genus *Halomonas.* Numbergiven above each bar indicates number of species coded respective gene.

**Additional file 6: Table S1.** Supplementary data for Radar Chart.

## Data Availability

Data is available within this manuscript. Sequences were downloaded from NCBI database. Gene bank accession numbers: AB242910, AB680702, AB680891, AB681733, AB681766, AB971837, AF054286, AF211860, AF211861, AF212202, AF212204, AF212206, AF212218, AF251143, AF465604, AJ271864, AJ306893, AJ320530, AJ417388, AJ427627, AJ431369, AJ515365, AJ564880, AJ640133, AJ876733, AM229314, AM229315, AM229316, AM229317, AM238662, AM941388, AY245449, AY268080, AY382579, AY671975, AY858696, AY962236, AY962237, DQ131909, DQ421808, DQ645593, DQ834966, DQ836238, EF117909, EF121853, EF121854, EF144147, EF144148, EF144149, EF421176, EF442769, EF527873, EF613112, EF613113, EF622233, EU085033, EU135707, EU159469, EU218533, EU305728, EU305729, EU373088, EU447162, EU447163, EU541349, EU557315, EU822512, EU909458, FJ429198, FJ984862, GCA_011742165, GCF_000409775, GCF_000759345, GCF_001662285, GCF_002879615, GCF_002930105, GCF_003045775, GCF_003056305, GCF_003202165, GCF_003254665, GCF_004363555, GCF_004551485, GCF_004798965, GCF_005780185, GCF_006540005, GCF_007989625, GCF_008274785, GCF_013697085, GCF_014192055, GCF_014192215, GCF_014192275, GCF_014651875, GCF_900100195, GCF_900100755, GCF_900108215, GCF_900116705, GCF_900142755, GQ232738, GQ281062, GQ354374, GU726750, HE661586, HM026177, HM242216, JF766572, JN242765, JQ716246, JQ762286, JQ762289, JQ781698, JX870002, KC237714, KF010830, KF479230, KF963827, KM066108, KP259554, KP301091, KR024741, KT796562, KU221020, KU320882, KU886576, KX008964, KX090359, KX953854, KY034384, KY034386, KY034408, KY039330, LT223576, LT558840, M93354, M93355, M93358, MF782431, MF850257, MG030686, MH071180, MH071181, MH071182, MK138622, MK346303, MK347065, MK357745, MN099429, MN435603, MT180568, MT372904, MT759855, MT759856, MT759857, MT760065, MT760070, MT760104, MT760115, MT760136, NC_014532, NZ_CP013106, NZ_CP018139, NZ_CP021435, NZ_CP065435, SDSD01000014, X87217, X87218, X92150, X92417, X93493 and X93493 (National Center for Biotechnology Information, U.S. National Library of Medicine, https://www.ncbi.nlm.nih.gov/) [9].

## Abbreviations

CDS: Coding sequence/the coding region of the gene
DoeA: Ectoine hydrolase
DoeC: Aspartate-semialdehyde dehydrogenase DoeC in ectoine degradation (EC 1.2.1.11)
DoeX: DNA-binding protein DoeX, ectoine utilization regulator
EctC: l-Ectoine synthase (EC 4.2.1.108)
EctD: Ectoine hydroxylase
EutB: Ectoine utilization protein EutB, threonine dehydratase-like
EutC: Ectoine utilization protein EutC, similar to ornithine cyclodeaminase
ICNP: The International Code of Nomenclature of Prokaryotes
LPSN: List of Prokaryotic names with Standing in Nomenclature
MCL: The Markov Cluster Algorithm
ME: Minimum Evolution method
MEGA X: Molecular Evolutionary Genetics Analysis across computing platforms version 10.0
MEGA X: Molecular Evolutionary Genetics Analysis, Version 10
ML: Maximum-Likelihood method
NCBI: National Center for Biotechnology Information
NJ: Neighbor-Joining method
NMPDR: National Microbial Pathogen Database Resource
PLfams: Genus-specific families
RAST: Rapid Annotation using Subsystem Technology
rRNA: Ribosomal RNA
spp.: Species
TeaA: Ectoine/hydroxyectoine TRAP transporter substrate-binding periplasmic protein TeaA
TeaB: Ectoine/hydroxyectoine TRAP transporter small permease protein TeaB
TeaC: Ectoine/hydroxyectoine TRAP transporter large permease protein TeaC
UspA: Universal stress protein UspA in Ectoine TRAP cluster
