# Supplementary figures and images for: The diversity of unique 1,4,5,6-Tetrahydro-2-methyl-4-pyrimidinecarboxylic acid coding common genes and Universal stress protein in Ectoine TRAP cluster (UspA) in 32 Halomonas species
Source: BMC Res Notes. 2021 Aug 3;14:296. doi: 10.1186/s13104-021-05689-3 (PMC8330102; doi:10.1186/s13104-021-05689-3)

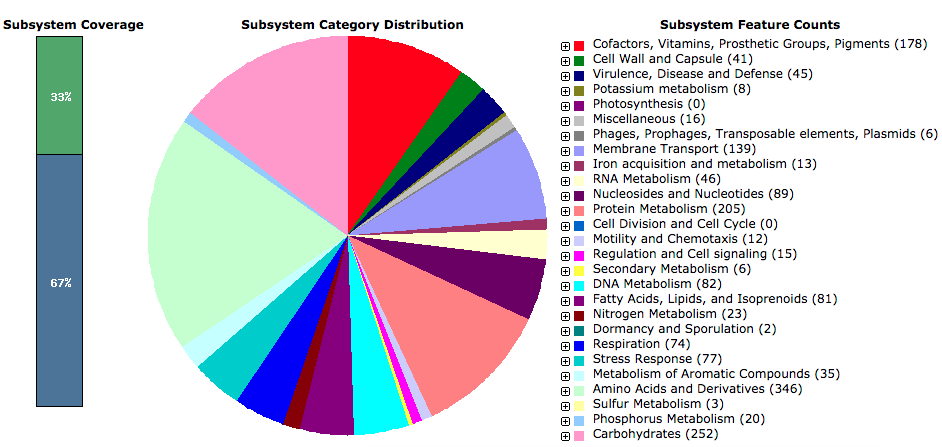

Supplement: Supplementary file 1 — Additional file 1: Figure S1. RAST genome analysis of H. elongata 1H9 indicates subsystem coverage and distributed subsystem features in annotated genome. Each subsystem feature possesses pathways encoded by respective genes. [file 13104_2021_5689_MOESM1_ESM.png]

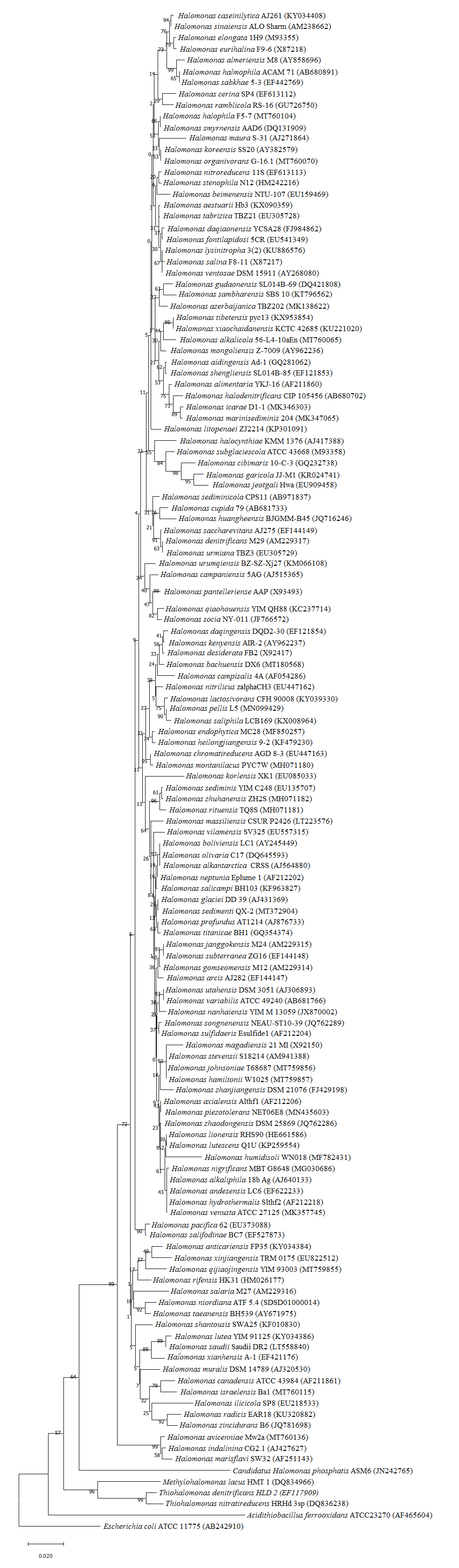

Supplement: Supplementary file 2 — Additional file 2: Figure S2. The evolutionary history of Halomonas species was inferred using the Neighbor-Joining method. Analysis using 16S rRNA gene sequences were conducted in MEGA X. The evolutionary distances were computed using the Jukes-Cantor method and are in the units of the number of base substitutions per site. The percentage of replicate trees in which the associated taxa clustered together in the bootstrap test (1000 replicates). [file 13104_2021_5689_MOESM2_ESM.tiff]

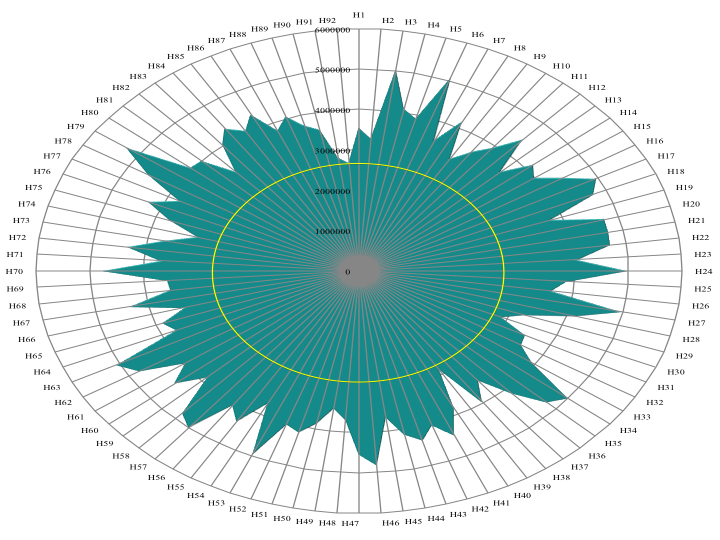

Supplement: Supplementary file 3 — Additional file 3: Figure S3. RADAR Chart of Genus Halomonas spp. (see supplementary table F1 for names of the species). Yellow circle indicates average genome length of each species and differences in genome length. [file 13104_2021_5689_MOESM3_ESM.png]

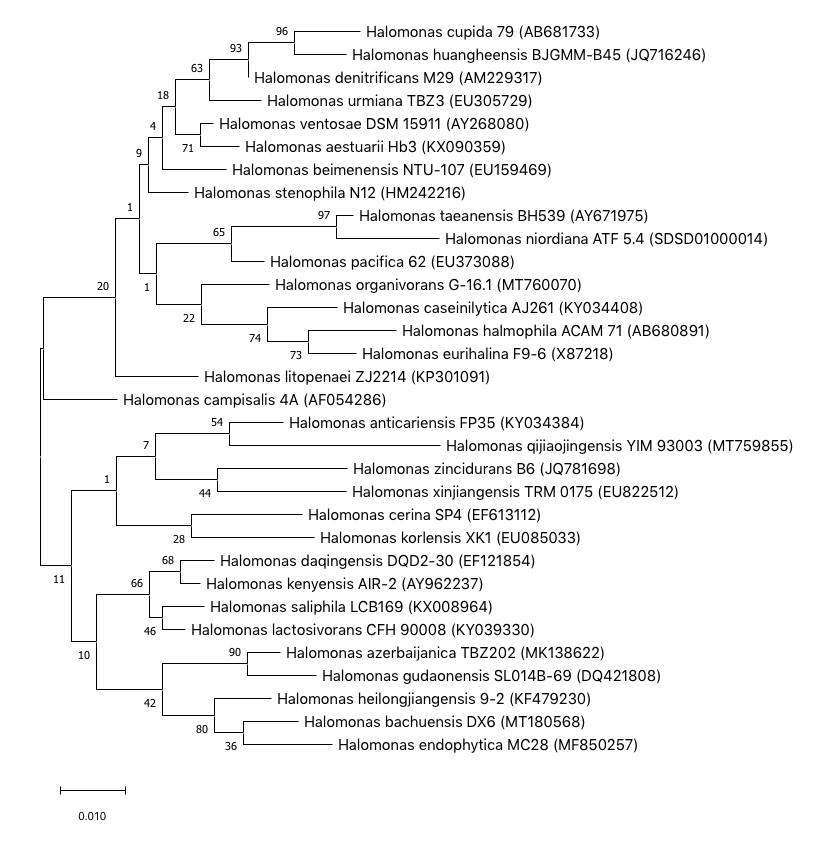

Supplement: Supplementary file 4 — Additional file 4: Figure S4. Maximum-likelihood analysis among Halomonas species was inferred from 16S rRNA gene sequences in MEGA X. The evolutionary distances were computed using the Jukes-Cantor method and are in the units of the number of base substitutions per site. The percentage of replicate trees in which the associated taxa clustered together in the bootstrap test (1000 replicates). [file 13104_2021_5689_MOESM4_ESM.png]

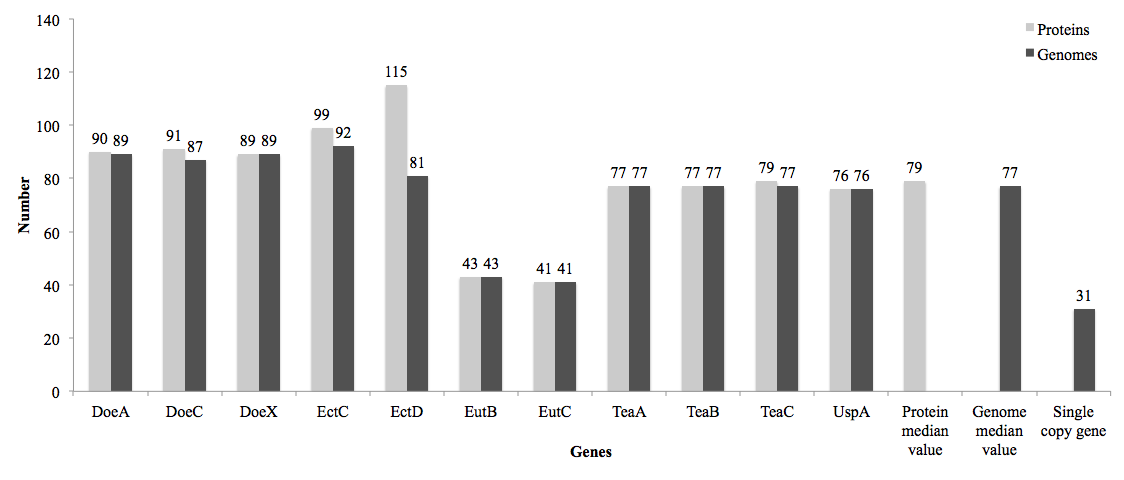

Supplement: Supplementary file 5 — Additional file 5: Figure S5. Single copy ectoine coding genes (DoeA-DoeC-DoeX-EctC-EctD-EutB-EutC-TeaA-TeaB-TeaC-UspA) in the genus Halomonas. Numbergiven above each bar indicates number of species coded respective gene. [file 13104_2021_5689_MOESM5_ESM.png]
